# Emergence and Genetic Variation of Neuraminidase Stalk Deletions in Avian Influenza Viruses

**DOI:** 10.1371/journal.pone.0014722

**Published:** 2011-02-23

**Authors:** Jinling Li, Heinrich zu Dohna, Carol J. Cardona, Joy Miller, Tim E. Carpenter

**Affiliations:** 1 Center for Animal Disease Modeling and Surveillance, University of California Davis, Davis, California, United States of America; 2 Department of Veterinary and Biomedical Sciences, College of Veterinary Medicine, University of Minnesota, St. Paul, Minnesota, United States of America; 3 National Center for Medical Intelligence, Fort Detrick, Maryland, United States of America; University of Oxford, Viet Nam

## Abstract

When avian influenza viruses (AIVs) are transmitted from their reservoir hosts (wild waterfowl and shorebirds) to domestic bird species, they undergo genetic changes that have been linked to higher virulence and broader host range. Common genetic AIV modifications in viral proteins of poultry isolates are deletions in the stalk region of the neuraminidase (NA) and additions of glycosylation sites on the hemagglutinin (HA). Even though these NA deletion mutations occur in several AIV subtypes, they have not been analyzed comprehensively. In this study, 4,920 NA nucleotide sequences, 5,596 HA nucleotide and 4,702 HA amino acid sequences were analyzed to elucidate the widespread emergence of NA stalk deletions in gallinaceous hosts, the genetic polymorphism of the deletion patterns and association between the stalk deletions in NA and amino acid variants in HA. Forty-seven different NA stalk deletion patterns were identified in six NA subtypes, N1–N3 and N5–N7. An analysis that controlled for phylogenetic dependence due to shared ancestry showed that NA stalk deletions are statistically correlated with gallinaceous hosts and certain amino acid features on the HA protein. Those HA features included five glycosylation sites, one insertion and one deletion. The correlations between NA stalk deletions and HA features are HA-NA-subtype-specific. Our results demonstrate that stalk deletions in the NA proteins of AIV are relatively common. Understanding the NA stalk deletion and related HA features may be important for vaccine and drug development and could be useful in establishing effective early detection and warning systems for the poultry industry.

## Introduction

Wild aquatic birds, such as *Anseriformes* (ducks and swans) and *Charadriiformes* (gulls and shorebirds) are reservoir hosts for avian influenza viruses (AIV) [1], [2], [3]. However, AIVs can cause outbreaks in poultry [4], [5], [6], [7], [8]. In some instances, AIV strains from poultry hosts have increased pathogenicity for poultry species [9], [10], and have acquired an ability to infect mammalian hosts [11], [12], [13], and/or have caused fatal infections in humans [14], [15]. Therefore, to minimize adverse effects in humans and poultry from AIV infections, it is important to understand what evolutionary changes occur in AIVs when they are transmitted from wild birds to poultry. Understanding these evolutionary changes can lead to better detection, prevention and control strategies.

AIVs interact with their hosts mostly through two glycoproteins, Hemagglutinin (HA) and Neuraminidase (NA). HA recognizes receptors on target cells and NA, a sialidase, assists virus entry and release [16], [17]. One observation that has been reported in viruses isolated during separate poultry outbreaks is a deletion in the stalk region of the NA [18], [19], [20], [21]. The stalk is a structure that separates the enzymatically and antigenically active “head” from the hydrophobic domain embedded in the viral membrane [22], [23], [24]. Little is known about the biological function of the NA stalk. Previous studies have shown that deletions in the NA stalk region influenced the virus' replication efficiency *in vivo*, increased its host range, reduced its NA enzymatic activity, and in some cases increased the virus' virulence [13], [25], [26]. Stalk deleted NAs (referred to as SΔNA hereafter) were reported sporadically in some AIV subtypes, e.g. H5N1, H6N1, H7N1, H7N3 and H9N2 [8], [9], [27], [28], [29]. SΔNA are often accompanied by observations of variants on the HA protein, such as the addition of glycosylation sites, presumably to maintain functional balance between HA and NA which is necessary for viral infectivity [30], [31], [32]. These HA variants could further influence viral antigenicity, virulence and pathogenicity [32], [33].

Although the SΔNA has been identified in different subtypes of poultry isolates, it is unclear whether there is a general correlation of SΔNAs with species in the order of *Galliformes* across different NA subtypes as claimed in previous publications [8], [9]. *Galliformes* (gallinaceous hosts) is an order of birds that includes important domestic and game birds, such as chickens, turkeys, pheasants, and quails. The aim of this study is to provide a broad understanding of the emergence of SΔNA through a comprehensive analysis of NA sequences. Our analysis showed SΔNA prevalence by subtypes, host and time period and demonstrated – with some exceptions – a general correlation between SΔNA and gallinaceous hosts. In addition, we analyzed the statistical correlation between SΔNA and variants on HA proteins.

## Results

### Occurrence of NA stalk deletion mutations

**Figure 1:**
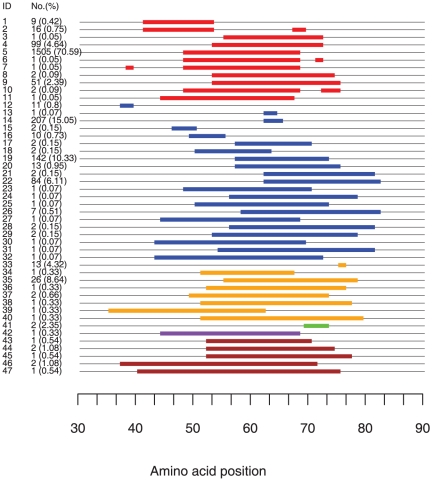
Patterns and prevalence of AIV SΔNA. Bars represent positions of missing amino acid residues. Colors indicate NA subtypes (red  =  N1, blue  =  N2, orange  =  N3, green  =  N5, purple  =  N6 and brown  =  N7). The first column on the left shows the deletion IDs assigned to each pattern. The second column shows the number of sequences having the corresponding deletion pattern and in parentheses the percentage of this pattern among all sequences of the same NA subtype. Percentages do not sum to 100 for the entire graph since they are calculated per NA subtype.

Among 4,920 neuraminidase sequences analyzed, 45.5% (2,238) carry stalk deletion mutations, i.e. 45.5% lost a stretch of amino acid residues in the stalk region (Table 1). The earliest SΔNA on record was from an H7N1 chicken-origin virus sampled in 1934 in Germany [34]. SΔNAs were observed with varying prevalence in six of nine NA subtypes (N1–N3 and N5–N7). Eighteen of 109 (16.5%) reported HA-NA subtypes in the public database (http://www.flu.lanl.gov/) contain SΔNAs. Eleven of these 18 (61%) HA-SΔNA subtypes include cases from poultry outbreaks as supported by publications [4], . Viruses from nine of 16 (56%) HA subtypes (H2–H7 and H9–H11) had SΔNA genes (Table 1). Forty-seven distinctive SΔNA patterns were identified among six NA subtypes with deleted portions ranging from one amino acid to 36 amino acid residues (Figure 1, Table S1). The only length polymorphism that we did not count as stalk deletion pattern was a Serine at position 40 among N5 sequences that was present in all North American isolates and with no counterpart amino acid in any Eurasian isolate.

**Table 1:** Occurrence of NA stalk deletions (SΔNA) among all reported avian influenza HA-NA^a^ subtypes.

	N1	N2	N3	N4	N5	N6	N7	N8	N9	N1–N9
**H1**	136^b^	5	7	-	5	4	+	-	4	161
**H2**	19	*1/11	42	1	2	1	3	4	18	1/101
**H3**	25	*1/110	9	2	9	48	3	239	3	1/448
**H4**	4	1/19	7	5	4	1/213	4	52	3	2/311
**H5**	*1526^c^/1665^b^	*97/212	*3/58	1	1	1	6	2	8	1626/1954
**H6**	*113/184	*20/122	5	2	2/17	10	+	44	3	135/387
**H7**	*49/71	*144/163	*30/127	6	1	2	4/54	4	4	227/432
**H8**	-	+	1	35	1	-	1	-	-	38
**H9**	9	*229/705	2	1	3	3	+	1	-	229/724
**H10**	5	2	12/19	4	3	6	3/110	6	2	15/157
**H11**	10	22	1/8	1	1	3	-	2	71	1/118
**H12**	1	+	1	3	36	-	-	-	3	44
**H13**	-	3	1	-	-	10	-	2	7	23
**H14**	-	-	-	-	2	-	-	-	-	2
**H15**	-	+	-	-	-	+	-	1	4	5
**H16**	-	-	10	-	-	-	-	-	1	11
**unknown**	3	1/1								1/4
**Total samples analyzed**	2,132/441^d^	1,375	297	61	85	301	181	357	131	4,920/3,229^d^
**Total # of SΔNA**	1,688/174^d^	494	46	0	2	1	7	0	0	2,238/724^d^
**SΔNA prevalence (%)**	79.17/39.45^d^	35.93	15.49	0	2.35	0.33	3.87	0	0	45.49/22.42^d^

a : hemagglutinin-neuraminidase.

b : Single number and the number behind slash denote the number of samples analyzed.

c : Number before slash denotes the number of sequences with SΔNA.

d : excluding sequences from HPAI H5N1 viruses.

-: No isolate has been reported for the subtype in publically accessible database, GenBank.

+:No samples were included due to unqualified sequences of the reported HA-NA subtype.

*: Some cases of the subtype were reported in poultry outbreaks.

The site of deletion and the frequency of each missing amino acid vary among NA subtypes. For each NA subtype, the most commonly dispensed positions are from 55 to 70 among all SΔNA subtypes except N5 (Figure S1).

### Distribution of NA stalk deletions

**Figure 2:**
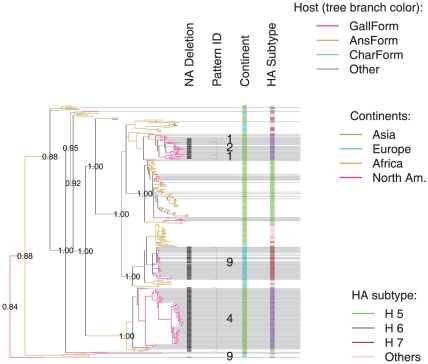
Phylogenetic tree of a subset of N1 sequences and distribution of SΔNA patterns (constructed in MrBayes 3.1.2). Values at nodes show estimated posterior probabilities for bipartitions. Branch colors indicate host order. Genes of isolates from gallinaceous hosts are shown by tree branches with extended grey lines. The first column from the left shows a black dash for each isolate with SΔNA. The second column shows the deletion pattern ID. Sequences with the same deletion pattern are denoted by square brackets numbered with pattern ID. The third column shows which continent each isolate was from and the fourth column indicates the HA subtype of each isolate.

The phylogenetic trees of NA nucleotides show that deletion patterns usually group into monophyletic clades that contain sequences belonging to a single deletion pattern (Figures 2, S2, S3, S4, S5 for N1, N2, N3 and N7 genes). Exceptions to monophyletic deletions are instances in which the same deletion pattern arose multiple times independently (deletion patterns 9, 12, 22, and 29, Figures 2, and S3), or patterns that are nested within a larger clade with a different deletion pattern (patterns 6, 7 and 10 within pattern 5, and pattern 17 within pattern 14). The patterns in the latter exception were most likely derived from existing patterns with additional deletions since they have the same deleted amino acid positions as those in the clade they are nested in (Figures 1, 2 and S3).

**Figure 3:**
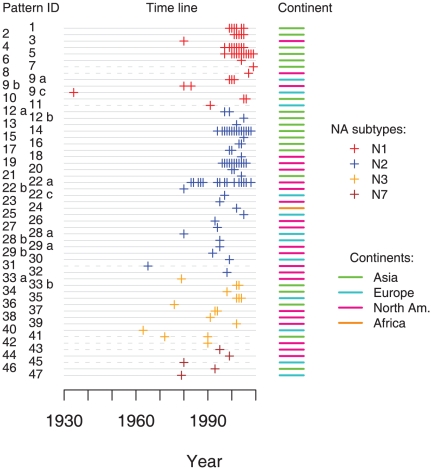
Spatial-temporal distribution of SΔNA patterns. Each line indicates an instance at which an SΔNA pattern emerged (refer to **Methods** **for definition). Pattern IDs on the left are the same as in Figure 1. Deletion patterns that are distributed over several distinct clades are indicated by lower case letters. Each cross indicates a year at which a particular pattern was observed. Crosses are color-coded for NA subtype. Bars on the right indicate the continent in which the deletion pattern was most commonly observed. Dotted time lines indicate deletion patterns that are not shown in the phylogenetic trees because their NA genes were partially sequenced.**

Most (38/47, 79%) of the SΔNA patterns were associated with a single HA subtype and a few patterns combined with multiple HA subtypes (9/47, 19%) (Table S1). Most mutants were limited to small geographic areas and a few patterns were found in isolates from multiple locations (10/47, 21%) (Table S1). The majority of SΔNAs (67%) persisted for less than a year (Figure 3). However, some patterns (4, 5, 14, 19, and 22) existed for many years (Figure 3, Table S1). Pattern 22 persisted for 25 years and appeared in both Asian and North American isolates (Figure 3 and S3).

### Gallinaceous hosts and NA stalk deletions

The percentage of SΔNA virus was higher among gallinaceous (1,355/1,955, 69%) than non-gallinaceous hosts (893/2,424, 36%), mainly in the orders *Charadriiformes* and *Anseriformes*. A high percentage of SΔNA mutants was observed among non-gallinaceous isolates in N1 deletions in the 2000s (804/1,086, 74%) due to the large number (∼800) of HPAIV H5N1 samples from infected wild birds. Apart from the HPAI H5N1 viruses, there were 97 SΔNA isolates from non-gallinaceous hosts. Among them, only two SΔNAs of the non-gallinaceous isolates had no obvious connection to domestic poultry. One was an H5N3 virus from a migrating mallard in Japan [45] and the other an H6N5 virus from a shearwater in Australia [46]. Most other SΔNAs of non-gallinaceous isolates were either from domestic birds or linked to poultry for the following reasons: (i) 33 were isolated from farm ducks [8], [47], [48], [49], (ii) ten were isolated from birds in live bird markets [50], [51], [52], (iii) 18 were isolated from hosts species that were not indigenous to the geographic region of the isolate, therefore, domestic, and (iv) the remaining 34 clustered within gallinaceous isolates in the phylogenetic tree.

Our analysis of the joint transition rates between all combinations of host types and NA states showed that estimated transition rates generally created a positive correlation between gallinaceous hosts and SΔNA. The rates leading to character combinations supporting a positive correlation (the combinations of gallinaceous/SΔNA and non-gallinaceous/full-length-NA) were subtracted from the rates leading to opposing character combinations (non-gallinaceous/SΔNA and gallinaceous/full-length-NA). A posterior distribution for this rate difference (*ΔQ*) was estimated. From this posterior distribution, a posterior probability that the rate difference exceeds zero was calculated (Table 2). A positive rate difference indicates that the positive correlation between gallinaceous hosts and SΔNA is due to uneven transition rates. For subclade 1 of N1, subclade 1 of N2 and N3, the posterior probabilities for a positive rate difference were greater than 0.95 (Table 2). However, this is not true for the following two groups, subclade 2 in N1 and subclade 2 in N2. Subclade 2 of N2, composed of N2 genes of H9N2 isolates of poultry outbreaks in China, includes 89% viruses from gallinaceous hosts but contains only 31% SΔNA mutants (Figure S3). The reverse is true for the HPAIV H5N1 subclade that contains 53% viruses from non-gallinaceous hosts yet has 99% SΔNA sequences (Figure S2).

**Table 2:** Bayesian estimation of joint transitions between gallinaceous and non-gallinaceous hosts and full-length-NA and SΔNA.

					Posterior probability that deletions are irreversible	
Subtype	Group^a^	Number of sequences analyzed	*ΔQ* mean (95% range)^b^	Posterior probability that *ΔQ*>0	Non- *Galliformes*	*Galliformes*
N1	Subclade 1 (not HP^c^ H5N1)	498	386 (316; 468)	1	0.04	0.92
	Subclade 2 (HP H5N1)	1291	225 (−890; 1400)	0.65	0.26	0.61
N2	Subclade 1 (not Eurasian H9N2)	592	425 (213; 567)	1	0	0.97
	Subclade 2 (Eurasian H9N2)	625	−13 (−199; 114)	0.12	0	0.98
N3	Entire tree	261	87 (0–513)	0.97	0.06	0.3

a : Analysis was performed separately for major clades when the trees were too large.

b : Difference of all rates that lead to the character combinations *Galliformes*/SΔNA and non-*Galliformes*/full-length-NA minus all rates that lead the character combinations non-*Galliformes*/SΔNA and *Galliformes*/full-length-NA. (refer to Methods).

c : highly pathogenic.

Models with a zero transition rate from deleted to full-length NA in *Galliformes* have high posterior probabilities among N1 and N2 sequences. This result implies that restoration of full-length-NA from SΔNA is unlikely for N1 and N2 in *Galliformes* (Table 2). These posterior probabilities are generally low in non-*Galliformes* (Table 2).

### Geographic area and NA stalk deletions

We tested for a correlation between SΔNA and Asia for N1, N2 and N3. In subclade 1 of N1 and in both N2 subclades, there is a significant positive correlation between SΔNA and an isolate being from Asia (Table 3). In subclade 2 of N1, the correlation is negative and no correlation was found for N3 (Table 3).

**Table 3:** Bayesian estimation of joint transitions between Asia and non-Asia and full-length-NA and SΔNA.

Subtype	Group^a^	*ΔQ* mean (95% range)^b^	Posterior probability that *ΔQ*>0
N1	Subclade 1 (not HP^c^ H5N1)	213 (145; 256)	1
	Subclade 2 (HP^c^ H5N1)	−1184 (−1515; −673)	0
N2	Subclade 1 (not Eurasian H9N2)	286 (170; 401)	1
	Subclade 2 (Eurasian H9N2)	771 (373; 1563)	1
N3	Entire tree	−207 (−511; 2)	0.19

a : Analysis was performed separately for major clades when the trees were too large.

b : Difference of all rates that lead to the character combinations Asia/SΔNA and non-Asia/full-length-NA minus all rates that lead the character combinations non-Asia/SΔNA and Asia/full-length-NA. (refer to Methods).

c : highly pathogenic.

### HA modifications and NA stalk deletions

**Figure 4:**
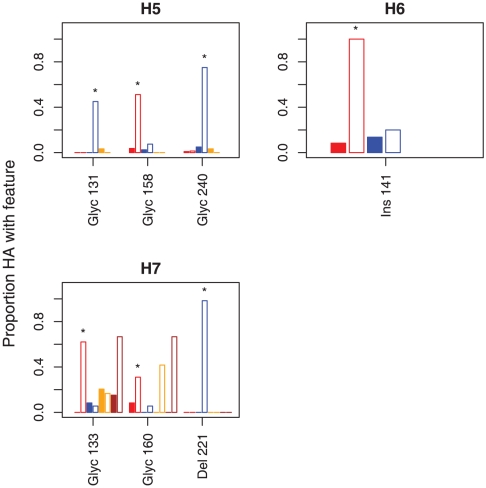
Proportions of HA protein features among viruses with full-length-NA (filled bars) or with SΔNA (open bars). The X-axis indicates the features, where Glyc denotes a glycosylation site, Del denotes a deletion, Ins denotes an insertion and the number indicates the amino acid position according to the H3 numbering. NA subtypes are color-coded (red  =  N1, blue  =  N2, orange  =  N3, brown  =  N7). Asterisks denote HA features whose SΔNA are statistically correlated with the HA feature since the posterior probability that *ΔQ* exceeds zero is greater than 0.95 (refer to Methods and Table 4).

To understand the potential impact of SΔNA on other viral properties, such as antigenicity, we tested whether any amino acid residues of HA protein features, such as glycosylation sites, are statistically correlated with the SΔNA genotype. Seven HA features were positively associated with SΔNA as indicated by the posterior probability of a positive rate difference (i.e. a rate difference that generates a positive correlation) exceeding 0.95. The features include five glycosylation sites, one deletion and one insertion that are identified in H5, H6 and H7 (Figure 4). All of these associations are NA-subtype-specific because no HA variant is significantly associated with SΔNA in more than one NA subtype (Figure 4, Table 4). The distribution of the seven features on the HA phylogenies is shown in the supplemental material (Figures S6, S7, S8 for H5–H7).

**Table 4:** Bayesian estimation of joint transitions between full-length NA and SΔNA and selected HA features.

Subtype	Number of sequences analyzed	Feature	Position	*ΔQ* mean (95% range)^a^	Posterior probability that *ΔQ*>0	Associated NA deletion patterns^c^
H5N1	119	Glycosylation	158	859 (30; 1776)	0.998	4, 5, 6, 11
H5N2	120	Glycosylation	131	546 (1; 1557)	0.978	25, 33
			240	136 (29; 425)	>0.999	20, 25, 30
H6N1	136	Insertion^b^	141	943 (212; 1293)	>0.999	1, 2, 4
H7N1	42	Glycosylation	133	520 (13; 1603)	0.987	9, 10
		Glycosylation	159^d^	749 (114; 1726)	0.995	9
H7N2	138	Deletion^b^	221	1545 (266; 3046)	0.991	21
		Glycosylation	160	499 (6; 1585)	0.955	39

a : Difference of all rates that lead to the character combinations feature present/SΔNA and feature absent/full-length-NA minus all rates that lead the character combinations feature absent/SΔNA and feature present/full-length-NA (refer to Methods).

b : Blanks in HA sequences that are associated with SΔNA are designated as deletions and blanks in HA sequences that are associated with full-length-NA are designated as insertions in HA sequences associated with SΔNA.

c : NA deletion pattern IDs that are associated with the HA feature that has a higher proportion among AIVs with SΔNA than among AIVs with full-length-NA.

d : There is no exact corresponding position on the H3 sequence. This position is on an insertion between position 159 and 160 on the H3 sequence.

## Discussion

In this study, we conducted a comprehensive analysis of the polymorphic AIV NA stalk regions using a large set of sequences of natural isolates (as opposed to laboratory-adapted isolates). Forty-seven different NA stalk deletion (SΔNA) patterns were identified in six NA subtypes, N1–N3 and N5–N7. An analysis that controlled for phylogenetic dependence due to shared ancestry showed SΔNA to be positively correlated with gallinaceous hosts and with some amino acid features on the HA. The analysis of the HA features estimated rates at which SΔNA were gained and lost on the HA tree depending on the presence of an HA feature. This analysis concerned only the overall rate of transitions and did not differentiate between transitions on the NA due to reassortment or due to mutation. It was therefore not necessary to estimate the reassortment rate between HA and SΔNA which is unknown for the analyzed dataset. Five glycosylation sites, one insertion and one deletion on the HA were identified to be statistically SΔNA-associated and all of these associations were specific to HA-NA subtype combinations. Our results further suggested that SΔNA mutations essentially cannot be restored to full-length in gallinaceous hosts. One challenge for our analysis was the unevenness of the sequence data due to varying sampling schemes. By fitting transition rates based on phylogenies, we could account for the uneven relatedness and the tendency of shared characteristics among closely related sequences. This was especially important for HPAIVs that are the source of a large number of very closely related sequences. However, when no SΔNAs were observed in non-gallinaceous hosts, the fitted transition rates could not distinguish between a restoration to a full-length NA stalk and an extinction of SΔNA mutants. Despite the limitation of our analysis, the findings shed light on the origin, spread and persistence of SΔNA as well as the functional balance between HA and NA [53].

The results of our analysis that controlled for phylogenetic dependence due to shared NA ancestry suggest that the positive correlation between SΔNA and gallinaceous hosts is caused by host-dependent transition rates between full-length-NA and SΔNA. Previous studies have indicated that naturally-occurring SΔNA mutants tend to appear in gallinaceous hosts but did not demonstrate a general correlation between host and SΔNA [8], [9], [28]. Laboratory studies showed that SΔNA mutations arose from duck-origin viruses after these viruses were passaged through gallinaceous hosts [13], [19], [54]. Our results provide further evidence that the processes demonstrated in laboratory studies generated the patterns observed in naturally-occurring viruses. The emergence of SΔNA in AIV infected gallinaceous hosts seems to be a general phenomenon that is widely detected in several NA subtypes.

However, the correlation between gallinaceous hosts and SΔNA mutations is not ubiquitous. In the N2 subclade 2, only 31% of the H9N2 isolates from poultry outbreaks were SΔNA mutants (Tables 2 and S1, Figure S3). As for the N1 subclade of HPAIV H5N1, almost all non-gallinaceous isolates had SΔNA (Figure S2). Besides the HPAIV H5N1 N1 subclade, there were 97 SΔNA sequences of other subtypes from non-gallinaceous isolates. Hence, SΔNA mutants appear to be generated in *Galliformes* but can be transmitted to other hosts. Whether the SΔNA of other subtypes can persist at low prevalence in non-gallinaceous birds is unknown. The high diversity of deletion patterns found in several NA subtypes suggests that these deletions convey a general advantage for the viruses in gallinaceous hosts that does not depend on the particular NA amino acid sequence. The fact that the same deletion pattern arose more than once in several instances indicates that the number of possible deletion patterns that confer this advantage is limited.

Our results showed a positive correlation between Asian locations and SΔNAs in three instances (both subclades of N2 and subclade 1 of N1). This correlation could be due to different farm practices in Asia (lower compliance with biosecurity measures and more mixed-species farms) and/or AIV sampling schemes biased towards poultry species in Asia. Among HP H5N1, there is a negative correlation between Asian locations and SΔNAs because all HP H5N1 that spread from Asia to other regions carried SΔNAs whereas within Asia two forms of NA (SΔNA and full-length-NA) of HP H5N1 co-existed (Figure S2).

This study also uncovered amino acid features on HA proteins that are statistically correlated with SΔNA. Previous studies demonstrated that shortened NA stalk length reduces NA activity [25] and that an efficient virus replication requires a matching reduction of HA activity [32], [55]. It has been shown for H1N1 and H7N1 that glycosylation sites on the HA globular head structure reduce HA affinity for receptor binding and make the virus less dependent on NA function [32], [56], [57], [58]. Viruses with these additional HA glycosylation sites replicated efficiently when combined with SΔNA and were less sensitive to NA inhibiting drugs [32], [33], [56], [57], [58]. In H7N1, the glycosylation site that conferred these effects was Asn149 [32], [33], [57] (or 133 according to the H3 numbering [59]), the same glycosylation site that is significantly correlated with SΔNA on N1 according to our results (Figure 4, Table 4). Our analysis showed other putative glycosylation sites in HA of other subtypes, as well as insertion and deletion that are statistically correlated with SΔNA (Figure 4, Table 4). Given the requirement of functional balance between HA and NA [32], [56], [57], [58], we speculate that the HA features identified by our methods could also reduce the HA affinity for host cell receptors and therefore, the virus' sensitivity to NA inhibitors. The fact that SΔNAs are widely associated with isolates of gallinaceous hosts while each HA feature occurs only with a single NA subtype could be an evidence that SΔNAs are adaptations to gallinaceous hosts whereas the accompanying HA features are secondary adaptations to SΔNAs. In theory, some of the correlations between HA features and SΔNAs could be the by-product of two pair-wise correlations with a third feature on an internal gene. Investigating such effects would require techniques to analyze multiple correlations while controlling for phylogenetic dependencies, which will be topics for future studies.

In summary, our results showed that SΔNAs are widely observed in AIVs. They are repeatedly associated with poultry outbreaks but are also found in non-poultry hosts. SΔNA mutants should be of special concern for the poultry industry since they could imply an adaptation of a virus to gallinaceous hosts. Previous researchers suggested that these viruses bear the risk of a pandemic [60], [61], [62]. AIV with SΔNA and SΔNA-associated HA features might be less sensitive to NA inhibiting drugs or reduce the efficacy of vaccines developed using a similar virus with full-length NA. Therefore, we believe it is important to closely monitor the emergence of SΔNA mutants in poultry and other species and prevent extended AIV circulation in poultry.

## Methods

### Sequence retrieval

AIV NA and HA nucleotide sequences and HA amino acid sequences were retrieved on December 17, 2009 from public influenza database (http://www.flu.lanl.gov/) using keywords type A and avian host. Nucleotide sequences of all lengths were retrieved while amino acid sequences were restricted to full length. NA nucleotide sequences were excluded from the analysis if the stalk region was not fully sequenced, i.e. if the first sequenced nucleotide position was after position 90 or the last sequenced position was before 270, counting adenine (A) in the start codon (ATG) as position 1. Furthermore, HA and NA sequences were excluded if they were acquired from viruses that were manipulated in laboratories (e.g. being expanded through laboratory animal or modified for the purpose of vaccine development), or if sequences were duplicates of an earlier submission of the same gene of the same strain, or erroneous as identified in a previous publication [63], or from non-avian isolates. A total of 4,920 NA, 5,596 HA nucleotide and 4,702 HA amino acid sequences were included in this study.

### Sequence alignment and identification of stalk deletions

All qualified nucleotide sequences of the same NA subtype were aligned using Clustal W (BioEdit v7.0.5, Ibis Therapeutics, Carlsbad, CA). All nucleotide sequences were translated into amino acid sequences within each subtype alignment using the same program. Deletion patterns in the stalk region (positions 30 to 90 for amino acids and 90 to 270 for nucleotides) were adjusted manually at both nucleotide and amino acid levels by moving nucleotides or amino acids between both positions that flank the deleted region. Nucleotides were adjusted to avoid stretches of missing nucleotides being flanked by incomplete codons. After translation, amino acids flanking a deletion were assigned to the flanking positions that best matched the consensus sequence. If permitted by the consensus sequences, deletion patterns were further adjusted to minimize the numbers of distinct deletion patterns. Deletion patterns were ordered by NA subtype and by size of the deleted region within each NA subtype. An identification (ID) number was assigned to each deletion pattern by numbering the ordered deletion patterns consecutively.

Phylogenetic trees were constructed for each NA subtype from aligned NA nucleotide sequences using weighted neighbor-joining (function *bionj* in the *R*-package *ape*) [64]. Trees were rooted with corresponding gene segments of the 1918 H1N1 human virus. The tree branches were labeled with deletion patterns. Due to their sizes, all trees are included in the supplemental material (Figures S2, S3, S4, S5). The first and last observation of each separate emerging SΔNA was recorded. Each different SΔNA pattern and distinct clades of the same SΔNA pattern were counted as separate emerging SΔNA. Two clades of the same SΔNA pattern were counted as distinct if they were separated by non-deleted sequences and had an estimated distance of more than 1% nucleotide changes between their nodes.

### Identification of HA features correlated with NA stalk deletion

HA nucleotide and amino acid sequences were aligned using MUSCLE with two alignment iterations [65], [66]. Aligned HA amino acid sequences, excluding positions within cleavage site, were screened for three possible features, namely putative glycosylation sites (NXT/S, where X could be any amino acid residues except Proline) [28], deletions or insertions. A script was written in *R* [67] that identifies the positions of all three features in all HA sequences (Script S1). HA and NA sequences were linked by strain names. HA and NA sequences with matching location, host species, serial number and year of sampling were assumed to be from the same isolate. The H3 numbering system was used for all HA subtypes as described in a previous study [59].

### Estimation of correlations between two characters

**Figure 5:**
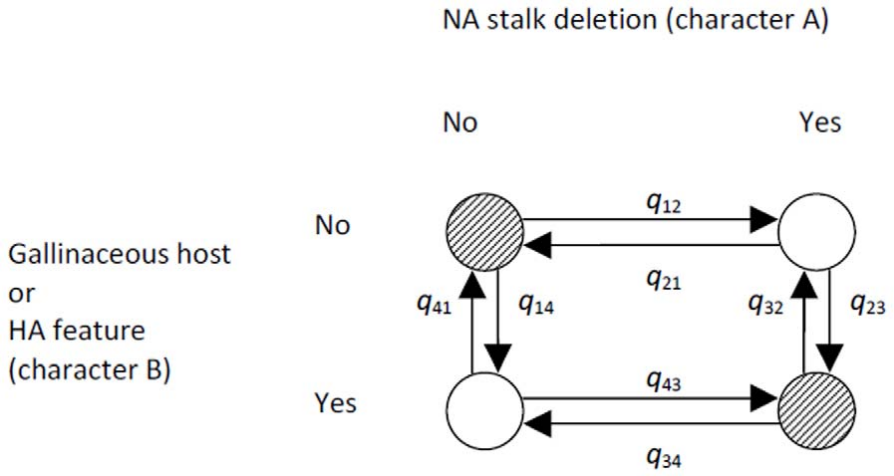
Illustration of all possible combinations of two binary characters (A and B) and the transition rates between these combinations. The model fits rates for the transitions per infinitely small time interval and therefore allows the change of only one state at a time (i.e. no rates are fitted for diagonal transitions). The shaded character combinations are expected to dominate if characters A and B are positively correlated. This positive correlation is created by transition rates if *ΔQ* = *q*_21_+*q*_23_+*q*_41_+*q*_43_−(*q*_12_+*q*_14_+*q*_32_+*q*_34_)>0.

We tested for correlations of SΔNA with host, with HA features and with geographic region while controlling for phylogenetic dependence due to shared NA or HA ancestry. The associations between NA stalk state and host or region were analyzed based on NA trees. The association between NA stalk state and HA features was analyzed based on HA trees. Characters of interest (i.e. state of NA stalk, host type, presence of HA feature and geographic region) were coded as binary characters and estimating the transition rates among the four possible combinations of two binary characters based on phylogenetic trees using the software package BayesTraits [68], [69] (Figure 5). Character A represents NA stalk state (with or without stalk deletion) and character B can be either host (gallinaceous or non-gallinaceous), HA amino acid feature (present or absent) or region (Asia or non-Asia). Independent character evolution would imply that the transition rates between two states of one character do not depend on the state of the other character.

For a given association between character A and B, there are two supporting and two opposing character state combinations described in Figure 5. For example, an association between gallinaceous hosts and SΔNAs is supported by the character combinations *Galliformes*/SΔNA and non-*Galliformes*/full-length-NA and opposed by non-*Galliformes*/SΔNA and *Galliformes*/full-length-NA. The difference between all rates leading to the two supporting character state combinations minus all rates leading to the opposing character state combinations, *ΔQ*, was calculated as a measure for whether the character correlation is generated by interdependent transition rates (Figure 5).

Using a Bayesian framework, posterior distributions of the transition rates were estimated given a distribution of phylogenetic trees. The distributions of phylogenetic HA and NA trees were estimated by fitting a general time reversible (GTR) model with gamma distributed rates to nucleotide sequences using MrBayes 3.1.2 [70]. The Markov chains for the trees were run for 3 Mio time steps with a burn-in period of 250,000 and trees were sampled every 2,000 steps leading to 1375 sampled trees per chain. Two independent chains were run for each tree and it was verified that the chains converged to the same tree by comparing the topological distances between trees within and between the chains. Each BayesTraits analysis sampled from a total of 2750 trees per subtype.

Analysis using large trees can lead to log-likelihood values of the transition model below the smallest number that the BayesTraits code can represent. To avoid such low log-likelihood values, sequences were divided into groups and a separate parameter estimation was performed per group. NA sequences were grouped into NA subtypes and N1 and N2 were divided into two subclades within each subtype. N1 sequences were distinguished into the subclade that contains all HPAIV H5N1 NAs or all other N1 sequences. N2 sequences were separated into those from isolates of H9N2 outbreaks in China and those from other isolates. For HA sequences, trees were constructed for each HA-NA subtype, e.g. a separate tree was constructed for HA sequences from H5N1 viruses and from H5N2 viruses. HA sequences were grouped in this fashion since HA trees (Figures S6,S7, S8) suggested that the association between HA features and SΔNA depended on NA subtype. The N1 and H5 trees of the HPAIV H5N1 clade were further reduced by randomly selecting a single sequence from all clades whose maximum distance from its basal node was less than 0.01. Representative sequences belonging to closely related branches were retained by this trimming method.

A reversible-jump Monte Carlo Markov Chain was used to simultaneously estimate the posterior distributions of transition rates and the posterior probabilities of constraints imposed on the transition rates [69]. A posterior distribution of *ΔQ* was calculated. A range containing 95% of *ΔQ* values and a posterior probability that *ΔQ* exceeds zero was determined from the posterior distribution. A high posterior probability of *ΔQ* exceeding zero was interpreted as evidence that observed positive correlations between characters were due to correlated transition rates and not simply due to shared ancestry.

The correlation between SΔNA and HA features were analyzed based on HA trees. HA trees were chosen because in that case SΔNA can be acquired or lost by mutation and/or reassortment. Hence the NA states are a more labile character on the HA tree than on the NA tree. Larger volatility of the NA state is more likely to reveal functional correlations if they are present. A single rate was fitted for each transition, without distinguishing whether transitions were due to mutations or reassortment.

## Supporting Information

Figure S1 Proportions of sequences with deleted amino acids at positions in the stalk region of AIV Neuraminidases.
(0.29 MB EPS)

Figure S2 Neighbor-joining tree of N1 sequences. Branch colors indicate host types, pink for *Galliformes*, yellow for *Anseriformes*, green for *Charadriiformes* and grey for birds from other orders. Genes of isolates from gallinaceous hosts are shown by tree branches with extended grey lines. The first column from the left shows a black dash for each isolate with SΔNA. The second column shows the deletion pattern ID. Sequences with the same deletion pattern are denoted by square brackets numbered with pattern ID. Deletion patterns that are nested within larger deletion pattern clades are indicated by deletion pattern IDs on the left side of the brackets. The third column indicates the HA subtype of each isolate. The fourth column shows the continent each isolated is from. The two N1 subclades are shown by brackets in the last column. The box indicates the part of the tree that is shown in Figure 2.
(0.63 MB EPS)

Figure S3 Neighbor-joining tree of N2 sequences. Symbol descriptions are the same as in Figure S2.
(0.49 MB EPS)

Figure S4 Neighbor-joining tree of N3 sequences. Symbol descriptions are the same as in Figure S2.
(0.27 MB EPS)

Figure S5 Neighbor-joining tree of N7 sequences. Symbol descriptions are the same as in Figure S2.
(0.24 MB EPS)

Figure S6 Neighbor-joining tree of H5 sequences. Branch colors indicate host types, pink for *Galliformes*, yellow for *Anseriformes*, green for *Charadriiformes* and grey for birds from other orders. Genes of isolates from gallinaceous hosts are shown in pink branches with extended grey lines. The columns from left to right show NA stalk state for each sequence (a pattern ID is given for each isolate with SΔNA), NA subtype, continent of origin and various HA protein features. NA subtypes and continents are color-coded. The presence of described HA features (Glyc  =  glycosylation, Del  =  deletion and Ins  =  insertion) are shown by black dashes.
(0.61 MB EPS)

Figure S7 Neighbor-joining tree of H6 sequences. Symbol descriptions are the same as in Figure S6. Strain names are shown.
(0.29 MB EPS)

Figure S8 Neighbor-joining tree of H7 sequences. Symbol descriptions are the same as in Figure S6. Strain names are shown.
(0.31 MB EPS)

Table S1 Summary of HA/NA subtypes, prevalence, deleted region, sampling location and time per deletion pattern.
(0.03 MB DOC)

Script S1 R code to identify glycosylation sites and deletions in amino acid sequences.
(0.00 MB TXT)

## References

[1] Olsen B, Munster VJ, Wallensten A, Waldenstrom J, Osterhaus AD (2006). Global patterns of influenza a virus in wild birds.. Science.

[2] Munster VJ, Fouchier RA (2009). Avian influenza virus: of virus and bird ecology.. Vaccine.

[3] Webster RG, Bean WJ, Gorman OT, Chambers TM, Kawaoka Y (1992). Evolution and ecology of influenza A viruses.. Microbiol Rev.

[4] Capua I, Marangon S (2000). The avian influenza epidemic in Italy, 1999-2000: a review.. Avian Pathol.

[5] Cheung CL, Vijaykrishna D, Smith GJ, Fan XH, Zhang JX (2007). Establishment of influenza A virus (H6N1) in minor poultry species in southern China.. J Virol.

[6] Dunn PA, Wallner-Pendleton EA, Lu H, Shaw DP, Kradel D (2003). Summary of the 2001-02 Pennsylvania H7N2 low pathogenicity avian influenza outbreak in meat type chickens.. Avian Dis.

[7] Hirst M, Astell CR, Griffith M, Coughlin SM, Moksa M (2004). Novel avian influenza H7N3 strain outbreak, British Columbia.. Emerg Infect Dis.

[8] Spackman E, Senne DA, Davison S, Suarez DL (2003). Sequence analysis of recent H7 avian influenza viruses associated with three different outbreaks in commercial poultry in the United States.. J Virol.

[9] Banks J, Speidel ES, Moore E, Plowright L, Piccirillo A (2001). Changes in the haemagglutinin and the neuraminidase genes prior to the emergence of highly pathogenic H7N1 avian influenza viruses in Italy.. Arch Virol.

[10] Senne DA, Panigrahy B, Kawaoka Y, Pearson JE, Suss J (1996). Survey of the hemagglutinin (HA) cleavage site sequence of H5 and H7 avian influenza viruses: amino acid sequence at the HA cleavage site as a marker of pathogenicity potential.. Avian Dis.

[11] Keawcharoen J, Oraveerakul K, Kuiken T, Fouchier RA, Amonsin A (2004). Avian influenza H5N1 in tigers and leopards.. Emerg Infect Dis.

[12] Belser JA, Blixt O, Chen LM, Pappas C, Maines TR (2008). Contemporary North American influenza H7 viruses possess human receptor specificity: Implications for virus transmissibility.. Proc Natl Acad Sci U S A.

[13] Hossain MJ, Hickman D, Perez DR (2008). Evidence of expanded host range and mammalian-associated genetic changes in a duck H9N2 influenza virus following adaptation in quail and chickens.. PLoS One.

[14] Claas EC, Osterhaus AD, van Beek R, De Jong JC, Rimmelzwaan GF (1998). Human influenza A H5N1 virus related to a highly pathogenic avian influenza virus.. Lancet.

[15] Fouchier RA, Schneeberger PM, Rozendaal FW, Broekman JM, Kemink SA (2004). Avian influenza A virus (H7N7) associated with human conjunctivitis and a fatal case of acute respiratory distress syndrome.. Proc Natl Acad Sci U S A.

[16] Naeve CW, Hinshaw VS, Webster RG (1984). Mutations in the hemagglutinin receptor-binding site can change the biological properties of an influenza virus.. J Virol.

[17] Palese P, Tobita K, Ueda M, Compans RW (1974). Characterization of temperature sensitive influenza virus mutants defective in neuraminidase.. Virology.

[18] Di Trani L, Bedini B, Cordioli P, Muscillo M, Vignolo E (2004). Molecular characterization of low pathogenicity H7N3 avian influenza viruses isolated in Italy.. Avian Dis.

[19] Li J, zu Dohna H, Anchell NL, Adams SC, Dao NT (2010). Adaptation and transmission of a duck-origin avian influenza virus in poultry species.. Virus Res.

[20] Liu J, Okazaki K, Ozaki H, Sakoda Y, Wu Q (2003). H9N2 influenza viruses prevalent in poultry in China are phylogenetically distinct from A/quail/Hong Kong/G1/97 presumed to be the donor of the internal protein genes of the H5N1 Hong Kong/97 virus.. Avian Pathol.

[21] Okamatsu M, Saito T, Yamamoto Y, Mase M, Tsuduku S (2007). Low pathogenicity H5N2 avian influenza outbreak in Japan during the 2005-2006.. Vet Microbiol.

[22] Skehel J (2009). An overview of influenza haemagglutinin and neuraminidase.. Biologicals.

[23] Els MC, Air GM, Murti KG, Webster RG, Laver WG (1985). An 18-amino acid deletion in an influenza neuraminidase.. Virology.

[24] Russell RJ, Haire LF, Stevens DJ, Collins PJ, Lin YP (2006). The structure of H5N1 avian influenza neuraminidase suggests new opportunities for drug design.. Nature.

[25] Castrucci MR, Kawaoka Y (1993). Biologic importance of neuraminidase stalk length in influenza A virus.. J Virol.

[26] Munier S, Larcher T, Cormier-Aline F, Soubieux D, Su B (2010). A genetically engineered waterfowl influenza virus with a deletion in the stalk of the neuraminidase has increased virulence for chickens.. J Virol.

[27] Campitelli L, Mogavero E, De Marco MA, Delogu M, Puzelli S (2004). Interspecies transmission of an H7N3 influenza virus from wild birds to intensively reared domestic poultry in Italy.. Virology.

[28] Guo YJ, Krauss S, Senne DA, Mo IP, Lo KS (2000). Characterization of the pathogenicity of members of the newly established H9N2 influenza virus lineages in Asia.. Virology.

[29] Lee MS, Chang PC, Shien JH, Cheng MC, Chen CL (2006). Genetic and pathogenic characterization of H6N1 avian influenza viruses isolated in Taiwan between 1972 and 2005.. Avian Dis.

[30] Liu C, Eichelberger MC, Compans RW, Air GM (1995). Influenza type A virus neuraminidase does not play a role in viral entry, replication, assembly, or budding.. J Virol.

[31] Lu B, Zhou H, Ye D, Kemble G, Jin H (2005). Improvement of influenza A/Fujian/411/02 (H3N2) virus growth in embryonated chicken eggs by balancing the hemagglutinin and neuraminidase activities, using reverse genetics.. J Virol.

[32] Wagner R, Wolff T, Herwig A, Pleschka S, Klenk HD (2000). Interdependence of hemagglutinin glycosylation and neuraminidase as regulators of influenza virus growth: a study by reverse genetics.. J Virol.

[33] Baigent SJ, McCauley JW (2003). Influenza type A in humans, mammals and birds: determinants of virus virulence, host-range and interspecies transmission.. Bioessays.

[34] Obenauer JC, Denson J, Mehta PK, Su X, Mukatira S (2006). Large-scale sequence analysis of avian influenza isolates.. Science.

[35] Cauthen AN, Swayne DE, Schultz-Cherry S, Perdue ML, Suarez DL (2000). Continued circulation in China of highly pathogenic avian influenza viruses encoding the hemagglutinin gene associated with the 1997 H5N1 outbreak in poultry and humans.. J Virol.

[36] Hoffmann E, Stech J, Leneva I, Krauss S, Scholtissek C (2000). Characterization of the influenza A virus gene pool in avian species in southern China: was H6N1 a derivative or a precursor of H5N1?. J Virol.

[37] Kinde H, Read DH, Daft BM, Hammarlund M, Moore J (2003). The occurrence of avian influenza A subtype H6N2 in commercial layer flocks in Southern California (2000-02): clinicopathologic findings.. Avian Dis.

[38] Woolcock PR, Suarez DL, Kuney D (2003). Low-pathogenicity avian influenza virus (H6N2) in chickens in California, 2000-02.. Avian Dis.

[39] Capua I, Mutinelli F, Marangon S, Alexander DJ (2000). H7N1 avian influenza in Italy (1999 to 2000) in intensively reared chickens and turkeys.. Avian Pathol.

[40] Marangon S, Bortolotti L, Capua I, Bettio M, Dalla Pozza M (2003). Low-pathogenicity avian influenza (LPAI) in Italy (2000-01): epidemiology and control.. Avian Dis.

[41] Cappucci DT, Johnson DC, Brugh M, Smith TM, Jackson CF (1985). Isolation of avian influenza virus (subtype H5N2) from chicken eggs during a natural outbreak.. Avian Dis.

[42] Choi YK, Ozaki H, Webby RJ, Webster RG, Peiris JS (2004). Continuing evolution of H9N2 influenza viruses in Southeastern China.. J Virol.

[43] Liu JH, Okazaki K, Shi WM, Wu QM, Mweene AS (2003). Phylogenetic analysis of neuraminidase gene of H9N2 influenza viruses prevalent in chickens in China during 1995-2002.. Virus Genes.

[44] Lee CW, Senne DA, Linares JA, Woolcock PR, Stallknecht DE (2004). Characterization of recent H5 subtype avian influenza viruses from US poultry.. Avian Pathol.

[45] Tsukamoto K, Ashizawa T, Nakanishi K, Kaji N, Suzuki K (2009). Use of reverse transcriptase PCR to subtype N1 to N9 neuraminidase genes of avian influenza viruses.. J Clin Microbiol.

[46] Blok J, Air GM (1982). Block deletions in the neuraminidase genes from some influenza A viruses of the N1 subtype.. Virology.

[47] Li C, Yu K, Tian G, Yu D, Liu L (2005). Evolution of H9N2 influenza viruses from domestic poultry in Mainland China.. Virology.

[48] Li KS, Xu KM, Peiris JS, Poon LL, Yu KZ (2003). Characterization of H9 subtype influenza viruses from the ducks of southern China: a candidate for the next influenza pandemic in humans?. J Virol.

[49] Zhang P, Tang Y, Liu X, Liu W, Zhang X (2009). A novel genotype H9N2 influenza virus possessing human H5N1 internal genomes has been circulating in poultry in eastern China since 1998.. J Virol.

[50] Li Y, Lin Z, Shi J, Qi Q, Deng G (2006). Detection of Hong Kong 97-like H5N1 influenza viruses from eggs of Vietnamese waterfowl.. Arch Virol.

[51] Liu M, He S, Walker D, Zhou N, Perez DR (2003). The influenza virus gene pool in a poultry market in South central china.. Virology.

[52] Zhou NN, Shortridge KF, Claas EC, Krauss SL, Webster RG (1999). Rapid evolution of H5N1 influenza viruses in chickens in Hong Kong.. J Virol.

[53] Liu C, Air GM (1993). Selection and characterization of a neuraminidase-minus mutant of influenza virus and its rescue by cloned neuraminidase genes.. Virology.

[54] Sorrell EM, Perez DR (2007). Adaptation of influenza A/Mallard/Potsdam/178-4/83 H2N2 virus in Japanese quail leads to infection and transmission in chickens.. Avian Dis.

[55] Mitnaul LJ, Matrosovich MN, Castrucci MR, Tuzikov AB, Bovin NV (2000). Balanced hemagglutinin and neuraminidase activities are critical for efficient replication of influenza A virus.. J Virol.

[56] Baigent SJ, Bethell RC, McCauley JW (1999). Genetic analysis reveals that both haemagglutinin and neuraminidase determine the sensitivity of naturally occurring avian influenza viruses to zanamivir in vitro.. Virology.

[57] Baigent SJ, McCauley JW (2001). Glycosylation of haemagglutinin and stalk-length of neuraminidase combine to regulate the growth of avian influenza viruses in tissue culture.. Virus Res.

[58] Mishin VP, Novikov D, Hayden FG, Gubareva LV (2005). Effect of hemagglutinin glycosylation on influenza virus susceptibility to neuraminidase inhibitors.. J Virol.

[59] Nobusawa E, Aoyama T, Kato H, Suzuki Y, Tateno Y (1991). Comparison of complete amino acid sequences and receptor-binding properties among 13 serotypes of hemagglutinins of influenza A viruses.. Virology.

[60] Butt KM, Smith GJ, Chen H, Zhang LJ, Leung YH (2005). Human infection with an avian H9N2 influenza A virus in Hong Kong in 2003.. J Clin Microbiol.

[61] Peiris M, Yuen KY, Leung CW, Chan KH, Ip PL (1999). Human infection with influenza H9N2.. Lancet.

[62] Subbarao K, Klimov A, Katz J, Regnery H, Lim W (1998). Characterization of an avian influenza A (H5N1) virus isolated from a child with a fatal respiratory illness.. Science.

[63] Li J, zu Dohna H, Miller J, Cardona CJ, Carpenter TE (2010). Identifying errors in avian influenza virus gene sequences and implications for data usage of public databases.. Genomics.

[64] Paradise E, Gentleman R, Hornik K, Parmigiani (2006). Analysis of Phylogenetics and Evolution with R. Use R!;.

[65] Edgar RC (2004). MUSCLE: a multiple sequence alignment method with reduced time and space complexity.. BMC Bioinformatics.

[66] Edgar RC (2004). MUSCLE: multiple sequence alignment with high accuracy and high throughput.. Nucleic Acids Res.

[67] Team RDC (2009). A Language and Environment for Statistical Computing.2.9.2 ed..

[68] Pagel M (1994). Detecting correlated evolution on phylogenies: A general method for the comparative analysis of discrete characters.. Proceedings of the Royal Society of London Series B Biological Sciences.

[69] Pagel M, Meade A (2006). Bayesian Analysis of Correlated Evolution of Discrete Characters by Reversible-Jump Markov Chain Monte Carlo.. Am Nat.

[70] Ronquist F, Huelsenbeck JP (2003). MrBayes 3: Bayesian phylogenetic inference under mixed models.. Bioinformatics.

