# Supplementary material for: Emergence and Genetic Variation of Neuraminidase Stalk Deletions in Avian Influenza Viruses
Source: PLoS One. 2011 Feb 23;6(2):e14722. doi: 10.1371/journal.pone.0014722 (PMC3044137; doi:10.1371/journal.pone.0014722)
Supplement: Table S1 — Summary of HA/NA subtypes, prevalence, deleted region, sampling location and time per deletion pattern. (0.03 MB DOC) [file pone.0014722.s009.doc]

Table S1. Summary of HA/NA subtypes, prevalence, deleted region, sampling location and time per deletion pattern

| total # of seq | Subtype | ID | # aa del | #of seq | Prevalence w/i subtype | 1st del pos | Last del pos | % in galli | % in Eurasia | HA  subtypes | Countries | Time |
| --- | --- | --- | --- | --- | --- | --- | --- | --- | --- | --- | --- | --- |
| 2132 | N1 | 1 | 12 | 9 | 0.42 | 42 | 53 | 100.00 | 100.00 | H6N1:9 | Taiwan:9 | 1999-2005 |
|  | N1 | 2 | 14 | 16 | 0.75 | 42-53 | 68-69 | 93.75 | 100.00 | H6N1:16 | Taiwan:16 | 2001-2005 |
|  | N1 | 3 | 17 | 1 | 0.05 | 56 | 72 | 100.00 | 0.00 | H6N1:1 | USA:1 | 1980 |
|  | N1 | 4 | 19 | 99 | 4.64 | 54 | 72 | 92.93 | 100.00 | H5N1:13, H6N1:86 | China:78, Hong Kong:18, Viet Nam:3 | 1997-2005 |
|  | N1 | 5 | 20 | 1505 | 70.59 | 49 | 68 | 48.70 | 100.00 | H5N1:1505 | Afghanistan:5, Belgium:1, Burkina Faso:4,  Cambodia:5, Cameroon:1, China:399,  Cote dIvoire:6, Croatia:1, Czech Republic:8,  Denmark:9, Egypt:72, France:13, Gaza Strip:5,  Germany:34, Ghana:3, Hong Kong:91,  Hungary:5, India:31, Indonesia:125, Iran:2, Iraq:1, Israel:7, Italy:4, Japan:11,  Kazakhstan:2, Kuwait:8, Laos:37,  Malaysia:3, Mongolia:17, Niger:1,  Nigeria:48, Pakistan:10, Russia:57,  Saudi Arabia:5, Slovakia:4, Slovenia:5,  South Korea:11, Spain:1, Sudan:6, Sweden:11,  Switzerland:12, Thailand:159, Turkey:1,  Ukraine:3, United Kingdom:3, Viet Nam:258 | 1997-2009 |
|  | N1 | 6 | 21 | 1 | 0.05 | 49-68 | 72 | 100.00 | 100.00 | H5N1:1 | Thailand:1 | 2004 |
|  | N1 | 7 | 21 | 1 | 0.05 | 39 | 49-68 | 100.00 | 100.00 | H5N1:1 | India:1 | 2009 |
|  | N1 | 8 | 21 | 2 | 0.09 | 54 | 74 | 100.00 | 0.00 | H5N1:2 | USA:2 | 2007 |
|  | N1 | 9 | 22 | 51 | 2.39 | 54 | 75 | 90.20 | 96.08 | H5N1:1, H6N1:1,  H7N1:49 | Canada:1, Germany:2, Italy:47, USA:1 | 1934-2001 |
|  | N1 | 10 | 23 | 2 | 0.09 | 49-68 | 73-75 | 100.00 | 100.00 | H5N1:2 | Indonesia:2 | 2005-2006 |
|  | N1 | 11 | 23 | 1 | 0.05 | 45 | 67 | 100.00 | 100.00 | H5N1:1 | United Kingdom:1 | 1991 |
| 1398 | N2 | 12 | 2 | 11 | 0.79 | 38 | 39 | 90.91 | 100.00 | H9N2:11 | China:5, Hong Kong:6 | 1997-2005 |
|  | N2 | 13 | 2 | 1 | 0.07 | 63 | 64 | 0.00 | 100.00 | H6N2:1 | China:1 | 2002 |
|  | N2 | 14 | 3 | 207 | 14.81 | 63 | 65 | 81.68 | 100.00 | H3N2:1, H5N2:1,  H6N2:2, H9N2:203 | China:182, Hong Kong:19, Japan:6 | 1994-2008 |
|  | N2 | 15 | 4 | 2 | 0.14 | 47 | 50 | 100.00 | 100.00 | H9N2:2 | Pakistan:2 | 2005-2005 |
|  | N2 | 16 | 6 | 10 | 0.72 | 50 | 55 | 100.00 | 100.00 | H9N2:10 | China:10 | 2003-2004 |
|  | N2 | 17 | 13 | 2 | 0.14 | 58 | 70 | 100.00 | 100.00 | H9N2:2 | China:2 | 1999-2000 |
|  | N2 | 18 | 13 | 2 | 0.14 | 51 | 63 | 100.00 | 0.00 | H5N2:2 | USA:2 | 2004 |
|  | N2 | 19 | 16 | 142 | 10.16 | 58 | 73 | 89.93 | 0.00 | H7N2:142 | USA:142 | 1996-2006 |
|  | N2 | 20 | 18 | 13 | 0.93 | 58 | 75 | 100.00 | 0.00 | H6N2:13 | USA:13 | 2000-2004 |
|  | N2 | 21 | 19 | 2 | 0.14 | 63 | 81 | 50.00 | 100.00 | H5N2:1, H6N2:1 | France:1, Taiwan:1 | 2002-2004 |
|  | N2 | 22 | 20 | 84 | 6.01 | 63 | 82 | 97.62 | 23.81 | H4N2:1, H5N2:81,  H6N2:1, N2:1 | El Salvador:2, Guatemala:4, Italy:1, Japan:16,  Mexico:41, Taiwan:3, USA:17 | 1980-2008 |
|  | N2 | 23 | 22 | 11 | 0.79 | 57 | 78 | 100.00 | 0.00 | H2N2:11 | USA:11 | 1994-1997 |
|  | N2 | 24 | 22 | 1 | 0.07 | 49 | 70 | 100.00 | 100.00 | H6N2:1 | South Africa:1 | 2002 |
|  | N2 | 25 | 23 | 1 | 0.07 | 51 | 73 | 100.00 | 100.00 | H5N2:1 | Italy:1 | 2005 |
|  | N2 | 26 | 24 | 7 | 0.50 | 59 | 82 | 71.43 | 0.00 | H5N2:7 | USA:7 | 1993 |
|  | N2 | 27 | 24 | 1 | 0.07 | 45 | 68 | 100.00 | 0.00 | H7N2:1 | USA:1 | 1994 |
|  | N2 | 28 | 25 | 2 | 0.14 | 57 | 81 | 50.00 | 100.00 | H5N2:1, H9N2:1 | Germany:1, Italy:1 | 1980-1995 |
|  | N2 | 29 | 25 | 2 | 0.14 | 54 | 78 | 100.00 | 0.00 | H5N2:2 | USA:2 | 1992-1995 |
|  | N2 | 30 | 26 | 1 | 0.07 | 44 | 69 | 100.00 | 100.00 | H5N2:1 | Belgium:1 | 1999 |
|  | N2 | 31 | 27 | 1 | 0.07 | 55 | 81 | 100.00 | 0.00 | H6N2:1 | Canada:1 | 1965.00 |
|  | N2 | 32 | 29 | 1 | 0.07 | 44 | 72 | 100.00 | 0.00 | H7N2:1 | USA:1 | 1998 |
| 297 | N3 | 33 | 1 | 13 | 4.38 | 76 | 76 | 7.69 | 92.31 | H10N3:12, H5N3:1 | China:12, USA:1 | 1979-2003 |
|  | N3 | 34 | 16 | 1 | 0.34 | 52 | 67 | 100.00 | 100.00 | H7N3:1 | Pakistan:1 | 1998 |
|  | N3 | 35 | 23 | 26 | 8.75 | 56 | 78 | 100.00 | 100.00 | H7N3:26 | Italy:26 | 2002-2004 |
|  | N3 | 36 | 24 | 1 | 0.34 | 53 | 76 | 0.00 | 100.00 | H5N3:1 | Japan:1 | 1976 |
|  | N3 | 37 | 24 | 2 | 0.67 | 50 | 73 | 100.00 | 0.00 | H11N3:1, H7N3:1 | USA:2 | 1993-1994 |
|  | N3 | 38 | 26 | 1 | 0.34 | 52 | 77 | 100.00 | 0.00 | H7N3:1 | USA:1 | 1991 |
|  | N3 | 39 | 27 | 1 | 0.34 | 36 | 62 | 100.00 | 0.00 | H5N3:1 | USA:1 | 2002.00 |
|  | N3 | 40 | 28 | 1 | 0.34 | 52 | 79 | 100.00 | 100.00 | H7N3:1 | United Kingdom:1 | 1963 |
| 85 | N5 | 41 | 4 | 2 | 2.35 | 71 | 74 | 50.00 | 100.00 | H6N5:2 | Australia:1, Taiwan:1 | 1972-1990 |
| 301 | N6 | 42 | 24 | 1 | 0.33 | 45 | 68 | 100.00 | 0.00 | H4N6:1 | USA:1 | 1990 |
| 181 | N7 | 43 | 18 | 1 | 0.55 | 53 | 70 | 100.00 | 0.00 | H10N7:1 | USA:1 | 1995 |
|  | N7 | 44 | 22 | 2 | 1.10 | 53 | 74 | 100.00 | 0.00 | H10N7:2 | USA:2 | 1999 |
|  | N7 | 45 | 25 | 1 | 0.55 | 54 | 78 | NaN | 100.00 | H7N7:1 | Italy:1 | 1980 |
|  | N7 | 46 | 35 | 2 | 1.10 | 38 | 72 | 0.00 | 100.00 | H7N7:2 | Taiwan:2 | 1993 |
|  | N7 | 47 | 36 | 1 | 0.55 | 41 | 76 | 0.00 | 100.00 | H7N7:1 | Germany:1 | 1979 |
